# Plasma metabolomics of schizophrenia with cognitive impairment: A pilot study

**DOI:** 10.3389/fpsyt.2022.950602

**Published:** 2022-09-28

**Authors:** Yihe Jiang, Xiujia Sun, Miaowen Hu, Lei Zhang, Nan Zhao, Yifeng Shen, Shunying Yu, Jingjing Huang, Huafang Li, Wenjuan Yu

**Affiliations:** ^1^Shanghai Mental Health Center, Shanghai Jiao Tong University School of Medicine, Shanghai, China; ^2^Shanghai Pudong New Area Mental Health Center, School of Medicine, Tongji University, Shanghai, China; ^3^Shanghai Clinical Research Center for Mental Health, Shanghai, China; ^4^Shanghai Key Laboratory of Psychotic Disorders, Shanghai, China

**Keywords:** Schizophrenia, cognitive impairment, metabolomics, UPLC-MS/MS, biomarker

## Abstract

Schizophrenia (SCZ) acts as a complex and burdensome disease, in which the functional outcome can be validly predicted by cognitive impairment, as one of the core features. However, there still lack considerable markers of cognitive deficits in SCZ. Based on metabolomics, it is expected to identify different metabolic characteristics of SCZ with cognitive impairment. In the present study, 17 SCZ patients with cognitive impairment (CI), 17 matched SCZ patients with cognitive normal (CN), and 20 healthy control subjects (HC) were recruited, whose plasma metabolites were measured using ultra-high performance liquid chromatography-tandem mass spectrometry (UPLC–MS/MS). The result of metabolic profiling indicated the identification of 46 differentially expressed metabolites between HC, CN, and CI groups, with 7 differentially expressed metabolites between CN and CI groups. Four differential metabolites (imidazolepropionic acid, Homoserine, and Aspartic acid) were repeatedly found in both screenings, by which the formed biomarker panel could discriminate SCZ with cognitive impairment from matched patients (AUC = 0.974) and health control (AUC = 0.841), respectively. Several significant metabolic pathways were highlighted in pathway analysis, involving Alanine, aspartate and glutamate metabolism, D-glutamine and D-glutamate metabolism, and Citrate cycle (TCA cycle). In this study, several differentially expressed metabolites were identified in SCZ with cognitive impairment, providing novel insights into clinical treatment strategies.

## Introduction

Schizophrenia (SCZ) is a chronic and serious mental illness, usually running in families accompanied with some complex symptoms, such as delusions, hallucinations, disorganized speech, and so on (1). According to a systematic review, the prevalence of this disease is 4.6 per 1,000 persons globally (2). The abnormal manifestations and public stigma result in a huge burden on patients, accompanied with their families and society. Among the core symptoms of SCZ, cognitive impairment acts as the prime driver affecting the therapy and prognosis, predicting the disease development and prognostic global functional outcome in SCZ (3, 4). Cognitive impairment in SCZ is ubiquitous. Empirical reviews have consistently demonstrated that patients with SCZ generally exhibited significantly decreased cognitive performance in comparison with healthy individual, referring to working memory, attention, and processing speed (5). Meanwhile, this feature in SCZ is relatively stable and independent compared to the other core symptoms, which will not be appreciably aggravated or moderated with illness duration (6). With the generality and heterogeneity, early diagnosis and detection of cognitive dysfunction is critical for SCZ treatment. However, there still lack the accurate and reliable pathophysiological criteria for SCZ with cognitive impairment.

Metabolomics serves as a powerful technique for the comprehensive study of low molecular weight molecules or metabolites identified within cells and biological systems, which has been widely used in the discovery of psychiatric biomarkers with high accuracy, resolution, and sensitivity (7, 8). In studies related to SCZ, metabolomics has shown great potential in the etiology, diagnosis, and treatment. For example, in a recent study this technology was adopted was adopted to successfully select four metabolites of SCZ and establish a high accuracy diagnosis model, providing a valuable reference for early diagnosis and intervention (9). Besides, it is well-known that drug therapy is adopted as the main treatment for SCZ, but antipsychotic drugs produce significant metabolic side effects, involving weight gain, abnormal blood-lipid level, and so on. Metabolomics has also efficiently contributed to understanding the underlying mechanism of antipsychotic drugs in SCZ (10). Among the various types of biological samples applied in metabolomics, the blood sample is widely chosen with its convenience and less invasive, and has demonstrated excellent accuracy (9–12). Therefore, with the objective to search biomarkers of SCZ with cognitive impairment, the plasma metabolomics analysis was conducted on SCZ patients with different degrees of cognitive impairment and healthy individuals.

The separation, detection, and determination of metabolites are considered the core of metabolomics technology. Currently, the commonly used analytical platforms for metabolomics refer to liquid chromatography-mass spectrometry (LC-MS), gas chromatography-mass spectrometry (GC-MS), nuclear magnetic resonance (NMR), and enzyme assays (13). In recent years, ultra-high performance liquid chromatography-tandem mass spectrometry (UPLC–MS/MS) has displayed obvious advantages in sensitivity, rapidness, efficiency, and cost-saving, which increasingly applied in human plasma metabolomics research (12, 14). For example, a previous study demonstrated the high accuracy of UPLC-MS/MS in detecting serum concentration in SCZ (12). Hence, UPLC-MS/MS was performed in the present study.

Taken together, we hypothesized that SCZ with cognitive impairment would exhibit different metabolic characteristics compared to SCZ with cognitive normality and healthy individual. In this study, plasma samples were collected from the three groups. Based on UPLC-MS/MS technology, we expected to screen out the primary plasma biomarkers in the SCZ with cognitive impairment and explore their potential pathophysiological mechanisms. These differentially expressed metabolites could contribute to providing novel insight for clinical diagnosis, intervention, and treatment of the SCZ with cognitive impairment.

## Materials and methods

### Participants

The patients of the present study were collected from An Observational Study on Atypical Antipsychotics Long-term Treatment Patients with Schizophrenia (SALT-C), a large sample, non-interventional and long-term prospective clinical study in China (15). The study was approved by the Institutional Review Board of Shanghai Mental Health Center, with the number of 2010-35, 2016-23. The study has been registered on Clinicaltrials.gov under the trial registration number NCT02640911. Written informed consent was obtained from all recruited participants and their guardians.

The diagnosis of all patients was performed based on the Structured Psychiatric Interview using the Diagnostic and Statistical Manual of Mental Disorders, Fourth Edition (DSM-IV). The severity of SCZ was measured by Positive and Negative Symptoms Scale (PANSS) (16). The Montreal Cognitive Assessment Scale (MoCA) was applied to assessing the cognitive performance, in which a score of 26 or above indicated normal cognitive function, while a score below 26 indicated impaired cognitive function (17). In the present study, the MoCA was measured at baseline and follow-up of 52 ± 2 weeks, based on which SCZ patients were categorized into the impaired cognitive group (MoCA < 26 at both time points, group CI) and the normal cognitive group (MoCA ≥ 26 at both time points, group CN). To adjust for the differences in baseline characteristics, the propensity score matching (PSM) was adopted to select matching subjects in the CI and CN groups. The exclusion criteria referred to any organic brain disorder, substance dependency, or other factors that would influence cognitive performance. In addition, the healthy control subjects were additionally recruited from the staff of the Shanghai Mental Health Center, who had no first-degree relative with psychiatric illness.

### Blood sample preparation

The blood samples were sampled from participants in a fasting state in the morning, and centrifuged at 2,000 *g* for 10 min. After separation, all plasma samples were stored at −80°C. Prior to metabolomic analysis, the plasma samples were pretreated with liquid-liquid extraction, which were thawed at 4°C to minimize sample degradation. Briefly, each sample was added ice cold methanol with partial internal standards. After vortex, centrifuge, and redissolve, the supernatant was frozen to dry for subsequent analysis.

### Metabolomic analysis

In this study, the Q300 Kit provided by Metabo-Profile (Shanghai, China) was used in metabolomics analysis. All targeted metabolites were quantitated by UPLC-MS/MS system (Waters Corp., Milford, MA, USA). For the high-performance liquid chromatography (HPLC), ACQUITY HPLC BEH C18 1.7 × 10^−6^ M VanGuard precolumn (2.1 × 5 mm) and ACQUITY HPLC BEH C18 1.7 × 10^−6^ M analytical column (2.1 × 100 mm) were adopted, with column temperature of 40°C and sample manager temperature of 10°C. The mobile phase was composed of 0.1% formic acid solution (A) and acetonitrile/IPA (70:30) (B). Gradient elution was performed as follows: 0–1 min (5% B), 1–11 min (5–78% B), 11–13.5 min (78–95% B), 13.5–14 min (95–100% B), 14–16 min (100% B), 16–16.1 min (100–5% B), 16.1–18 min (5% B). The flow rate was 0.40 ml/min with the injection volume of 5.0 μl. For mass spectrometer, capillary 1.5 (ESL+), 2.0 (ESL-) Kv, source temperature 150°C, de-solvation temperature 550 °C, and de-solvation gas flow 1,000 L/h.

### Statistical analysis

The raw data files generated by UPLC-MS/MS were processed using the TMBQ software (v1.0; Human Metabolomics Institute, Shenzhen, Guangdong, China), so as to perform peak integration, calibration, and calculate the concentration of each analyte in samples. Multivariate statistical analyses and univariate analyses were carried out with iMAP (v1.0) to identify differences between groups, composed of principal component analysis (PCA), partial least squares discriminant analysis (PLS-DA), and orthogonal partial least squares discriminant analysis (OPLS-DA). The variable significance in projection (VIP) generated in OPLS-DA processing served as the criterion for metabolites screening. Metabolites with VIP >1 and *p* < 0.05 (univariate analyses were based on whether the data were normally distributed) were considered the statistically significant differentially expressed metabolites. Binary logistic regression analysis was conducted to analyze the predictors of the differentially expressed metabolites. The receiver operating characteristic (ROC) curve was plotted based on the available data, and the area under the ROC curve (AUC) was used to evaluate the diagnostic capability of differentially expressed metabolites. Metabolic pathway analysis was performed using the *Homo sapiens* (Hsa) sets on the Kyoto Encyclopedia of Genes and Genomes (KEGG, http://www.kegg.jp). Pathway enrichment analysis was performed on MetaboAnalyst 4.0 (http://www.metaboanalyst.ca/MetaboAnalyst/). Other statistical analyses were performed using SPSS 23.00 statistical software. The data were expressed as the mean ± standard deviation (x ± SD), and analyzed by the one-way ANOVA test or the independent sample *t*-test. Differences were considered statistically significant at *p* < 0.05.

## Results

### Characteristics of participants

**Figure 1:**
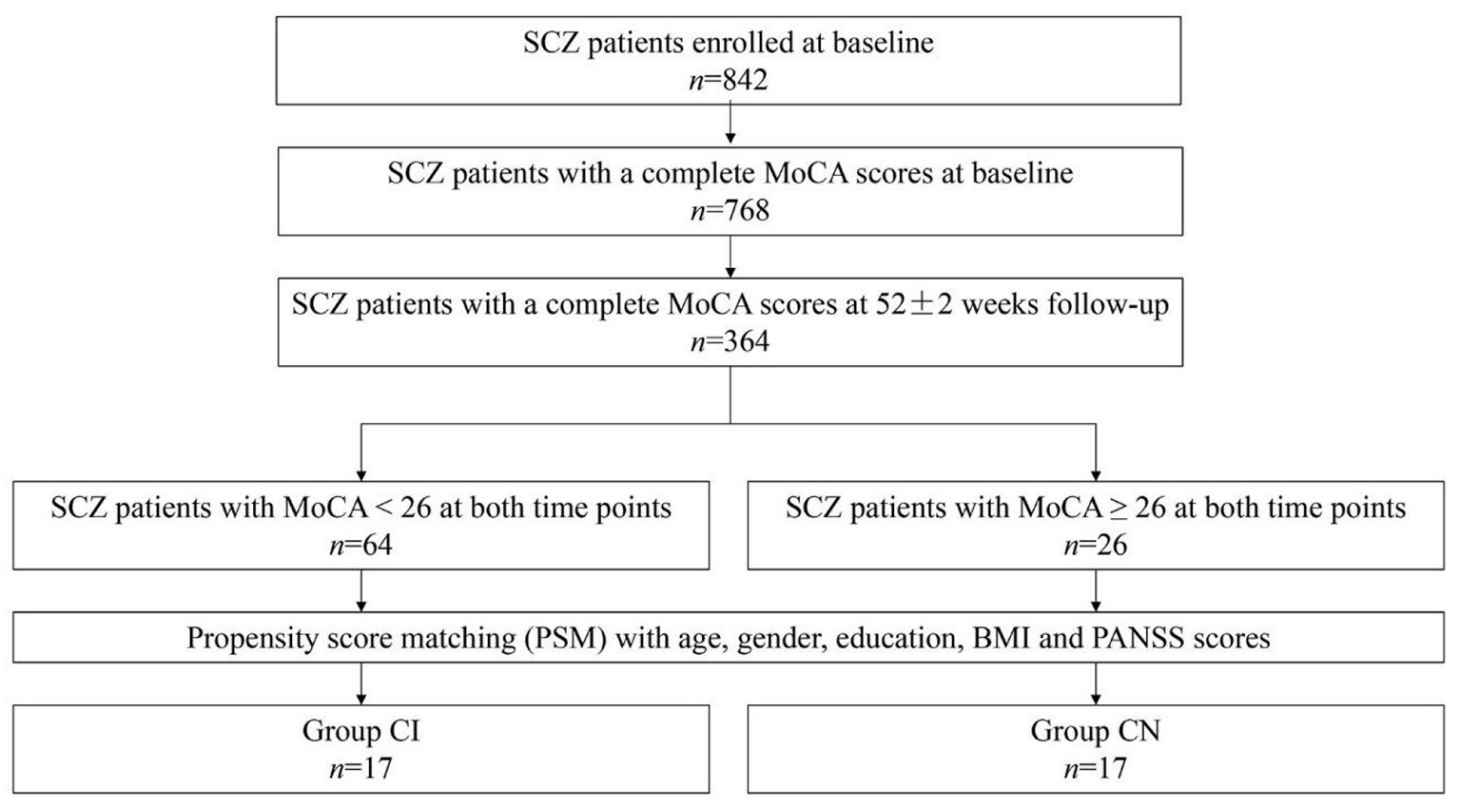
Study flow diagram. A total of 34 SCZ patients were screened from SALT-C in the present study.

A total of 842 SCZ patients were recruited for the SALT-C (15). After careful screening, 34 SCZ patients were enrolled in the present study eventually, covering SCZ patients with cognitive impairment (*n* = 17, it was shown that MoCA < 26 at both time points, group CI), and SCZ patients with cognitive normal (*n* = 17, MoCA ≥ 26 at both time points, group CN). The group CI and group CN were matched in age, gender, education, body mass index (BMI), and PANSS scores (*p* > 0.05), indicating the suitability for the comparison. The detailed screening process is presented in Figure 1. In addition, 20 healthy individuals were recruited as healthy control. The demographics and clinical characteristics of participants are listed in Table 1.

**Table 1:** Demographics and clinical characteristics of participants.

**Characteristics**	**CI (*n* = 17)**	**CN (*n* = 17)**	**HC (*n* = 20)**
Age (years)	54.18 ± 13.97	49.53 ± 12.17	32.05 ± 4.62
Gender	F = 5, M = 12	F = 6, M = 11	F = 7, M = 13
Education (years)	10.00 ± 3.72	11.41 ± 3.12	16.2 ± 3.75
BMI (kg/m^2^)	24.85 ± 3.76	23.53 ± 3.14	23.15 ± 3.17
PANSS	55.18 ± 10.51	56.59 ± 13.00	N/A
Smoker (*n*, %)	5 (29.4%)	4 (23.5%)	3 (15%)
MoCA	16.88 ± 4.47	27.59 ± 1.23	27.95 ± 1.23
**Types of antipsychotic drugs**			
Quetiapine (*n*, %)	1 (5.9%)	3 (17.6%)	N/A
Olanzapine (*n*, %)	8 (47.1%)	7 (41.2%)	N/A
Risperidone (*n*, %)	1 (5.9%)	3 (17.6%)	N/A
Aripiprazole (*n*, %)	6 (35.3%)	9 (52.9%)	N/A
Clozapine (*n*, %)	7 (41.2%)	0	N/A
Amisulpride (*n*, %)	1 (5.9%)	0	N/A

CI, SCZ patients with cognitive impairment; CN, SCZ patients with cognitive normal; HC, health control. BMI, body mass index; PANSS, the total score of Positive and Negative Syndrome Scale; MoCA, the total score of Montreal Cognitive Assessment Scale. No PANSS score and drug are used in the healthy control group (N/A). Values are expressed as mean ± standard deviation.

### Discovery of discriminatory metabolites

**Figure 2:**
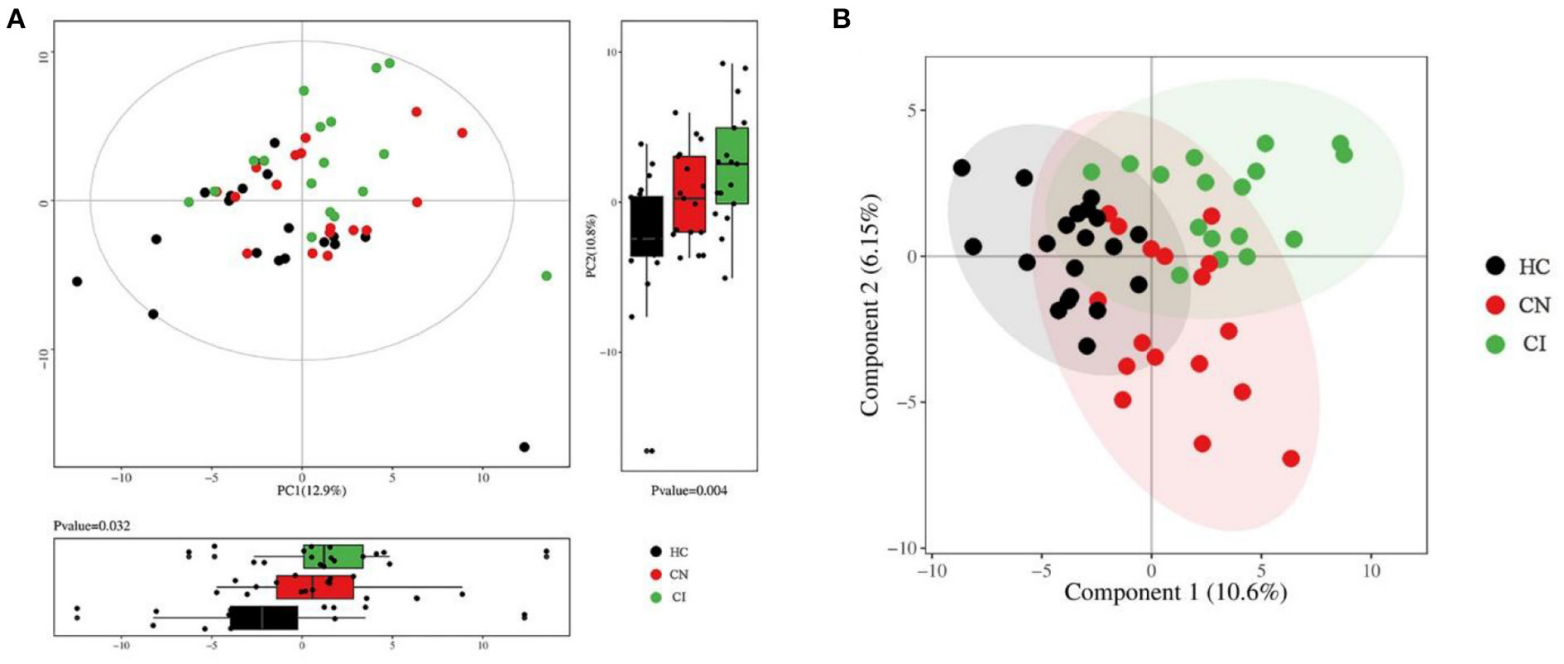
Multivariate statistical analysis of plasma metabolomics. **(A)** Overview of PCA score plots with principal component boxplot obtained from all samples. **(B)** Overview of PLS-DA score plots obtained from all samples.

After sampling and processing the plasma metabolite, 168 kinds of metabolites were successfully detected in each sample. The relative abundance of each metabolite class is depicted in Supplementary Figure 1. For the multivariate analyses, the PCA and PLS-DA were performed to explore the separation of metabolites between the three groups, obtaining a relatively clear classification, as shown in Figure 2. To visually illustrating the patterns of differences between groups, three groups of samples were pairwise compared by OPLS-DA. Scores plots and permutation plots of HC *vs*. CI and HC *vs*. CN demonstrated a robust quality for statistical models establishment (HC *vs*. CI, R^2^ = 0.863, Q^2^ = 0.644; HC *vs*. CN, R^2^ = 0.818, Q^2^ = 0.438), but lower quality parameters in model CN *vs*. CI (R^2^ = 0.771, Q^2^ = −0.128) (Supplementary Figure 2).

**Figure 3:**
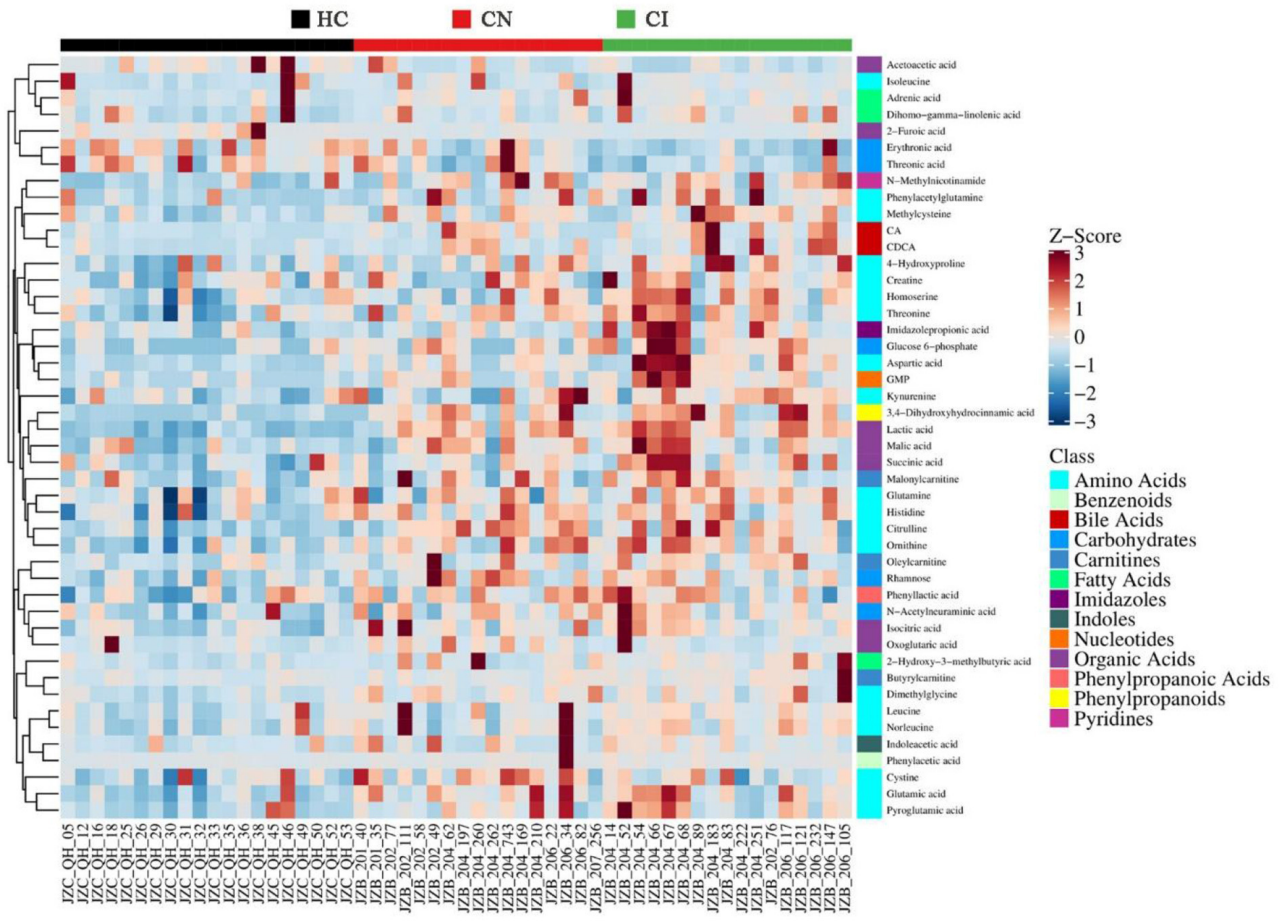
Heat map of the 46 differentially expressed metabolites between HC, CN and CI groups. The blue band indicates a decreased level of metabolite, and the red band indicates an increased level of metabolite.

Subsequently, the screening of potential biomarkers was carried out based on multi-dimensional statistics with VIP > 1 and univariate statistics with *p* < 0.05. With the comparison among the three groups, 46 differentially expressed metabolites were screened out (Supplementary Table 1), with the heatmap shown in Figure 3. To further explore the potential cognitive biomarkers, the identification of differences between group CN and CI was performed. Given the dissatisfying model of CN *vs*. CI, the univariate analyses were involved for further reference, and 7 differentially expressed metabolites were screened successfully (Table 2). Among them, Imidazolepropionic acid, Erythronic acid, Homoserine, and Aspartic acid also displayed significant differences in the previous comparison of three groups, which showed great potential as biomarkers for the development of cognitive dysfunction.

**Table 2:** List of differentially expressed metabolites between CN and CI.

**Metabolite**	**Class**	**CN (mean ±SD)**	**CI(mean ±SD)**	**Value of *p***
Imidazolepropionic acid	Imidazoles	0.7649 ± 0.0127	0.7914 ± 0.0335	1.45E-02
Erythronic acid	Carbohydrates	2.1629 ± 2.0511	1.0533 ± 1.6254	1.91E-02
Homoserine	Amino acids	6.0262 ± 2.1035	7.7902 ± 2.3761	2.61E-02
Glycolic acid	Organic acids	6.0910 ± 2.5021	4.2823 ± 2.2919	3.53E-02
Azelaic acid	Fatty acids	0.3199 ± 0.2840	0.1452 ± 0.1681	4.26E-02
Aspartic acid	Amino acids	6.2202 ± 4.3561	14.4624 ± 12.5125	4.57E-02
Malonic acid	Organic acids	2.9702 ± 0.1890	2.8591 ± 0.1186	4.83E-02

Mean represents the average relative abundance of metabolites in different groups. SD represents standard deviation. T-test was adopted for univariate analysis, and *p* < 0.05 is considered significant.

### ROC curve analysis

**Figure 4:**
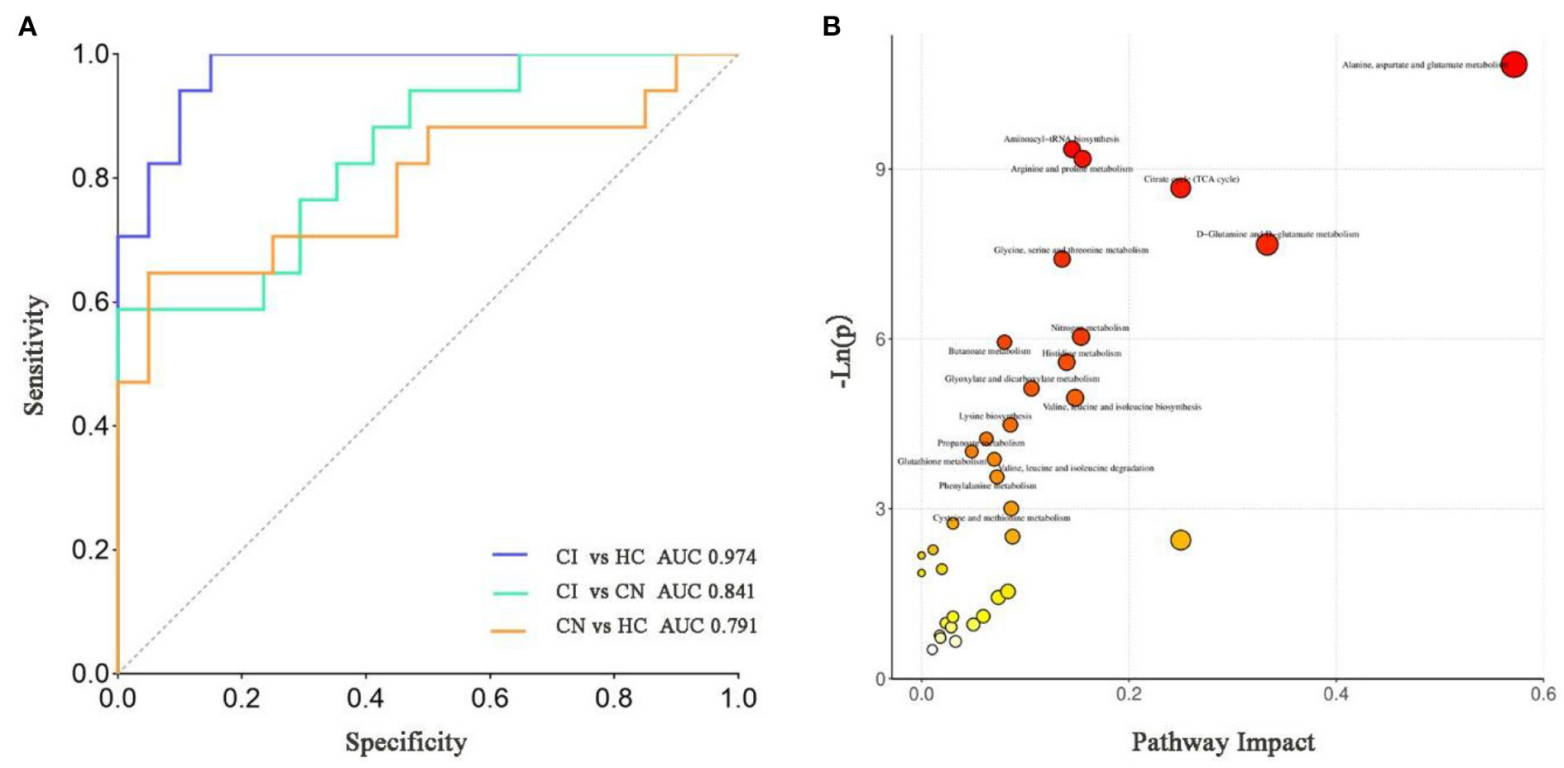
**(A)** The ROC diagnostic analysis of biomarker panel (Imidazolepropionic acid, Erythronic acid, Homoserine, and Aspartic acid) for different groups. **(B)** Pathway analysis bubble plot by the Hsa set in KEGG. The x-axis is the pathway impact, indicating the importance of differentially expressed metabolites in metabolic pathways. The y-axis is the negative logarithm of *p*-value. The size of pathway symbols represents the statistical significance level of pathway analysis. The color of pathway symbols represents the impact factor. Large sizes and dark colors represent central pathway enrichment and high pathway impact values, respectively.

ROC curve analysis was carried out to evaluate the diagnostic capability of differentially expressed metabolites. In order to obtain a potentially simplified biomarker panel, the binary logistic regression analysis was applied to the four differentially expressed metabolites. As depicted in Figure 4A, the CI could be accurately distinguished from HC or CN by the biomarker panel (Imidazolepropionic acid, Erythronic acid, Homoserine, and Aspartic acid), in which the AUC were 0.974 and 0.841, respectively. Consistently, the biomarker panel also could distinguish CN from HC (AUC = 0.791).

### Metabolic pathway analysis

Pathway analysis performed on the 46 differentially expressed metabolites by KEGG database, and a total of 35 pathways were enriched (Figure 4B). Based on *p*-value and pathway impact scores, the top three pathways were highlighted, referring to Alanine, aspartate and glutamate metabolism (*p* < 0.0001, impact = 0.57), D-glutamine and D-glutamate metabolism (*p* = 0.000467, impact = 0.33), and Citrate cycle (TCA cycle) (*p* = 0.000172, impact = 0.25).

## Discussion

Metabolomics has certain reliability in exploring plasma metabolites, through which the previous studies have demonstrated the metabolic variation in SCZ (11, 18). To our knowledge, plasma metabolite of cognitive function has been rarely reported, which, however, is a potentially stable and reliable predictor of long-term outcomes in SCZ. In this study, we explored the characteristic of plasma metabolism in SCZ with cognitive impairment based on UPLC–MS/MS combined with both univariate statistical methods and multivariate statistical methods. According the results of metabolic profiling, we identified 46 differentially expressed metabolites between HC, CN, and CI groups, and 7 differentially expressed metabolites in comparison between CN and CI groups. These results verify the advantages of metabolomics in searching differentially expressed metabolites.

Different kinds of plasma metabolites were identified in SCZ with cognitive impairment. In comparisons of three groups (46, HC *vs*. CN *vs*. CI) and two groups (7, CN *vs*. CI), four plasma metabolites appeared repeatedly, that the Imidazolepropionic acid, Homoserine, and Aspartic acid were significantly increased in CI, and Erythronic acid significantly decreased in CI. ROC analysis demonstrated a good discriminative power of the biomarker panel formed by the four metabolites in cognitive function in SCZ.

Among the four metabolites, aspartic acid is reported to be strongly associated with psychiatric disorders (19, 20), which has previously been demonstrated in the metabolomics study of SCZ (11). The study using LC/MS/MS-based approach found that aspartic acid can serve as a good biomarker to distinguish SCZ patients from healthy controls. Despite the absence of consideration of cognitive symptoms in the previous study, the consistent screening results validated the feasibility of aspartic acid as a biomarker in SCZ. However, the previous study showed lower aspartic acid levels in SCZ patients (11), which is inconsistent with our result, where the plasma aspartic acid level was higher in SCZ patients, especially in SCZ patients with cognitive impairment. It can result from by two factors. First, the uptake of aspartic acid by the brain from plasma has been demonstrated when plasma level remains high for relatively long periods (21). While high content of aspartic acid can be a toxin, which induces hyperexcitability of neurons, eliciting degeneration of neurons with excess, which will ultimately develop to cognitive impairment (22). Another, current research has suggested the accelerative role of aspartate metabolism in the inflammatory responses (23). Related studies generally have pointed out that the pathogenesis of cognitive impairment in SCZ is closely related to the abnormal immune system (24). As mentioned above, aspartic acid has the potential to recognize SCZ and the cognitive function of SCZ, but requiring further studies.

Additionally, aspartic acid can be converted into homoserine by a two-reduction step of the terminal carboxyl group. A previous study found both the increased metabolites in the serum of patients with Alzheimer's disease and Parkinson's disease, showing a tandem variation (25). Not surprisingly, our results also indicated the increased homoserine in SCZ with cognitive impairment, as with aspartic acid. In addition, to our knowledge, imidazolepropionic acid and erythronic acid have not been found in previous metabolomics studies of mental disorders, of which the underlying mechanism requires to be further studied. Anyway, our results illustrate that the down-regulation of erythronic acid and the up-regulation of imidazolepropionic acid, homoserine, and aspartic acid may be the causes of cognition decline of CI.

In addition to the screened differentially expressed metabolites, abnormal metabolic pathways also serve as a key point reflecting the pathology of cognitive impairment. The results of pathway analysis indicated the association of cognitive performance of SCZ with Alanine, aspartate and glutamate metabolism, D-glutamine and D-glutamate metabolism, as well as Citrate cycle (TCA cycle). Among them, Alanine, aspartate and glutamate metabolism, D-glutamine and D-glutamate metabolism suggest a critical role of glutamate and glutamine in this study. Previous studies have consistently suggested that the occurrence and development of SCZ is closely related to glutamate, forming the glutamatergic dysfunction hypothesis of SCZ (26). In the hypothesis, the dysregulation of γ-aminobutyric acid (GABA) level and N-methyl-D-aspartate (NMDA) receptor elicit the abnormality of brain glutamate concentration, which are considered the core of the pathogenic trigger of SCZ and symptoms (e.g., cognitive impairment) (27, 28). The abnormal elevated blood glutamate levels have been found in SCZ (29, 30) and the glutamate is related to cognitive function in SCZ (31), which is consistent with our results (Supplementary Table 1) and confirmed to the glutamatergic dysfunction hypothesis. However, the variations of glutamatergic metabolite levels in SCZ, covering its association with SCZ at different levels of cognitive function, remain ambiguous and require further investigation (28, 31).

In this study, the other important pathway, Citrate cycle (TCA cycle) was found promoted in SCZ and associated with cognitive impairment in SCZ, which support the possibility that abnormalities in energy metabolism contribute to SCZ (32). To be specific, the hypothesis holds that TCA cycle affects inflammatory response and induces blood-brain barrier damage, which lead to the occurrence and development of SCZ and the core symptoms (33–35). Indeed, it has been found that the TCA cycle was abnormal in SCZ compared with HC, which is consistent with our results (33). While the correlation between TCA cycle and the cognitive level of SCZ still requires further study.

Finally, our study has points of strengths and limitations that warrant discussion. The primary strength is that the propensity score matching in the real-world setting compensates for the lack of a randomized-controlled study. Another evident strength points to the performance of UPLC–MS/MS-based metabolite profiling, which is in high selectivity, reliability, and sensitivity. However, the major limitation of this study is the relatively limited sample size, that only 64 or 26 of the 364 patients were consistent low or high MoCA, possibly resulting from the long interval between baseline and follow-up (52 ± 2 weeks) in our study, compared with 48 ± 32.2 days in the previous study, where the validity of MoCA in SCZ and the stability of cognitive function in SCZ have been confirmed (36). The time interval is a key factor for future research to consider. Second, although we used PSM to balance out some differences in CI and CN, there still leave some between-group differences and within-group differences failing to be ruled out (e.g., drugs, individual underlying diseases, and individual dietary differences). A more rigorous experimental design could be considered in future studies. Additionally, our study is a preliminary exploratory study that requires further external validation.

## Conclusion

In summary, we demonstrated that the SCZ with cognitive impairment had a significant imbalance of metabolites. According the results of metabolic profiling, we identified 46 differentially expressed metabolites between HC, CN and CI groups, and 7 differentially expressed metabolites compared to CN and CI groups. Four differential metabolites (imidazolepropionic acid, Homoserine, and Aspartic acid) were repeatedly identified in both screenings, by which the formed biomarker panel could be accurately discriminate the SCZ with cognitive impairment from matched patients and healthy subjects. The metabolomics based on UPLC–MS/MS method provide an objective reference for clinical treatment strategies.

## Data availability statement

The original contributions presented in the study are included in the article/Supplementary material, further inquiries can be directed to the corresponding authors.

## Ethics statement

The studies involving human participants were reviewed and approved by Institutional Review Board of Shanghai Mental Health Center affiliated with Shanghai Jiao Tong University School of Medicine. The patients/participants provided their written informed consent to participate in this study.

## Author contributions

YJ: data analysis and manuscript writing. XS: identification and quantification of plasma metabolites. XS, HL, and WY: conception and design of the study. JH: ethical review. MH, LZ, and NZ: project implementation and data collection. HL and WY: critical reading of the manuscript. YS, SY, JH, HL, and WY: overall supervision of the project. All authors contributed to the manuscript and approved the submitted version.

## Funding

This study was supported by the Shanghai Clinical Research Center for Mental Health (19MC1911100), Collaborative Innovation Center for Translational Medicine at Shanghai Jiao Tong University School of Medicine (TM202016), and Sanming Project of Medicine in Shenzhen (No. SZSM202011014).

## Conflict of interest

The authors declare that the research was conducted in the absence of any commercial or financial relationships that could be construed as a potential conflict of interest.

## Publisher's note

All claims expressed in this article are solely those of the authors and do not necessarily represent those of their affiliated organizations, or those of the publisher, the editors and the reviewers. Any product that may be evaluated in this article, or claim that may be made by its manufacturer, is not guaranteed or endorsed by the publisher.

## Supplementary material

Supplementary Figure 1 The overview of identified metabolite classification classes. **(A)** Pie plot showing proportion of identified metabolite classes in all samples. **(B)** Stacked bar chart showing relative abundance of each metabolite classes in different groups.

Supplementary Figure 2 The OPLS-DA model building and validation. **(A–C)** OPLS-DA score plots, and **(D–F)** permutation plot between every two groups (HC vs. CI, HC vs. CN, and CN vs. CI).

Supplementary Table 1 List of differentially expressed metabolites between HC, CN and CI groups.
