# Supplementary figures and images for: Plasma metabolomics of schizophrenia with cognitive impairment: A pilot study
Source: Front Psychiatry. 2022 Sep 28;13:950602. doi: 10.3389/fpsyt.2022.950602 (PMC9554540; doi:10.3389/fpsyt.2022.950602)

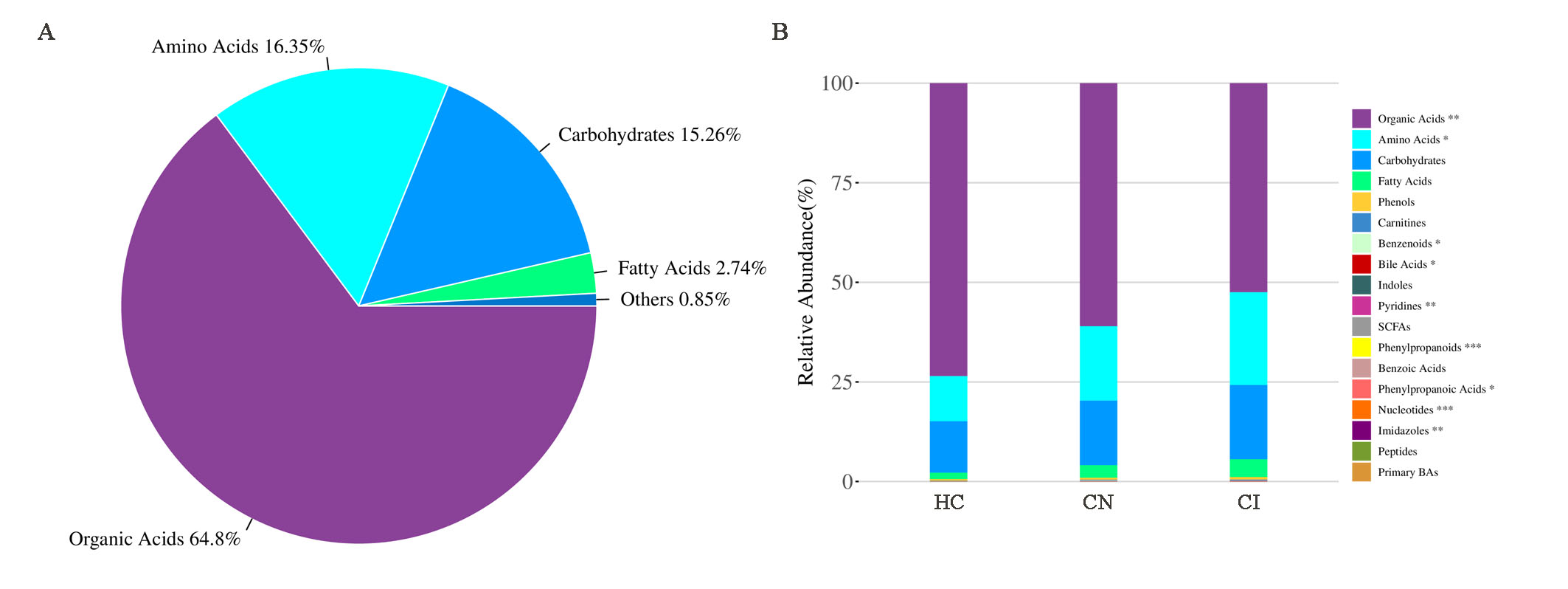

Supplement: Supplementary Figure 1 — The overview of identified metabolite classification classes. (A) Pie plot showing proportion of identified metabolite classes in all samples. (B) Stacked bar chart showing relative abundance of each metabolite classes in different groups. [file Image_1.JPEG]

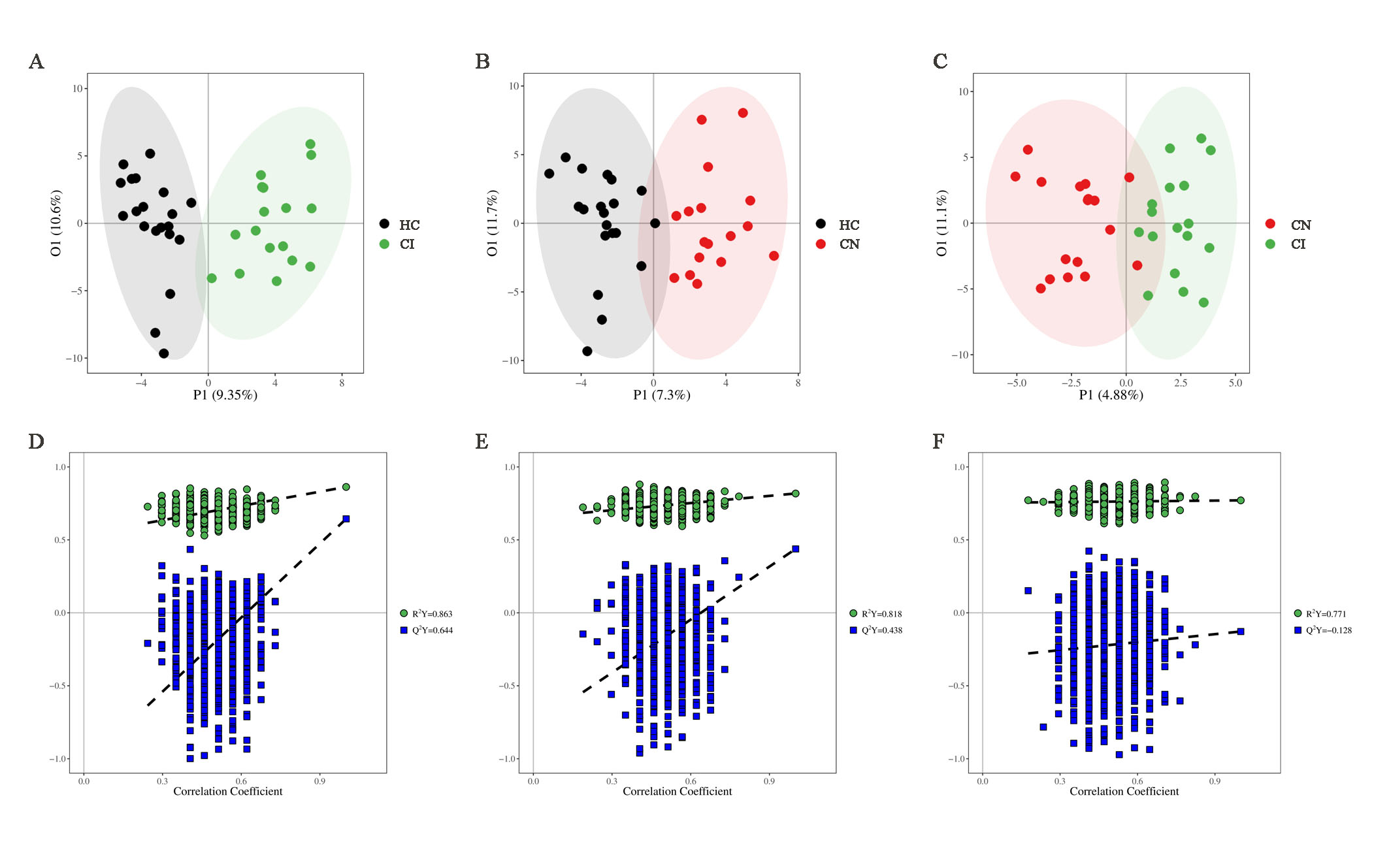

Supplement: Supplementary Figure 2 — The OPLS-DA model building and validation. (A–C) OPLS-DA score plots, and (D–F) permutation plot between every two groups (HC vs. CI, HC vs. CN, and CN vs. CI). [file Image_2.JPEG]
