# Supplementary material for: Plasma metabolomics of schizophrenia with cognitive impairment: A pilot study
Source: Front Psychiatry. 2022 Sep 28;13:950602. doi: 10.3389/fpsyt.2022.950602 (PMC9554540; doi:10.3389/fpsyt.2022.950602)
Supplement: Supplementary Table 1 — List of differentially expressed metabolites between HC, CN and CI groups. [file Table_1.docx]

| **Supplementary Table 1.** List of differentially expressed metabolites between HC, CN and CI groups. | | | | | | | |
| --- | --- | --- | --- | --- | --- | --- | --- |
| NO. | Metabolite | Class | HC (mean±SD) | CN (mean±SD) | CI(mean±SD) | Value of *p* | FDR |
| 1 | Lactic acid | Organic Acids | 525.2822±223.0073 | 1492.2415±548.4447 | 1793.5309±543.0010 | 2.71E-08 | 4.55E-06 |
| 2 | 3,4-Dihydroxyhydrocinnamic acid | Phenylpropanoids | 0.2731±0.4134 | 1.9359±1.6484 | 2.5650±2.0236 | 7.49E-06 | 6.29E-04 |
| 3 | Citrulline | Amino Acids | 41.1531±12.0395 | 73.5139±24.0725 | 72.9667±31.4448 | 5.32E-05 | 2.24E-03 |
| 4 | **Aspartic acid** | Amino Acids | 2.1655±2.2894 | 6.2202±4.3561 | 14.4624±12.5125 | 5.34E-05 | 2.24E-03 |
| 5 | Ornithine | Amino Acids | 75.7185±30.5535 | 120.9328±41.0653 | 126.8250±40.8530 | 1.48E-04 | 4.98E-03 |
| 6 | Glutamic acid | Amino Acids | 21.6098±16.4135 | 43.4600±22.2678 | 44.5212±22.5854 | 1.78E-04 | 4.98E-03 |
| 7 | GMP | Nucleotides | 0.1930±0.2639 | 0.5485±0.4769 | 1.1715±1.2545 | 2.26E-04 | 5.41E-03 |
| 8 | CA | Bile Acids | 0.1705±0.1879 | 0.7463±0.8974 | 1.3850±1.9901 | 5.45E-04 | 1.14E-02 |
| 9 | Glucose 6-phosphate | Carbohydrates | 0.5547±0.5474 | 1.0392±0.7331 | 1.6732±1.1171 | 6.53E-04 | 1.22E-02 |
| 10 | CDCA | Bile Acids | 0.3615±0.5065 | 1.2730±1.4220 | 2.7680±3.8478 | 1.01E-03 | 1.70E-02 |
| 11 | Dimethylglycine | Amino Acids | 3.9695±1.6528 | 7.2099±3.2291 | 8.7884±10.0255 | 1.30E-03 | 1.97E-02 |
| 12 | Threonine | Amino Acids | 80.5594±25.8703 | 104.4176±23.5333 | 113.0221±30.1655 | 1.41E-03 | 1.97E-02 |
| 13 | Acetoacetic acid | Organic Acids | 23409.0742±26912.8757 | 13392.1137±12915.2547 | 8330.0356±6114.3426 | 1.58E-03 | 2.04E-02 |
| 14 | Isocitric acid | Organic Acids | 3.4814±2.2885 | 7.0280±5.0751 | 7.4910±5.1333 | 1.93E-03 | 2.31E-02 |
| 15 | **Erythronic acid** | Carbohydrates | 2.4865±1.4678 | 2.1629±2.0511 | 1.0533±1.6254 | 2.16E-03 | 2.42E-02 |
| 16 | N-Methylnicotinamide | Pyridines | 3.6543±3.5619 | 7.2734±5.7688 | 8.3059±4.6218 | 2.87E-03 | 2.98E-02 |
| 17 | 2-Furoic acid | Organic Acids | 0.1757±0.4080 | 0.0460±0.0274 | 0.0356±0.0309 | 3.02E-03 | 2.98E-02 |
| 18 | Histidine | Amino Acids | 61.8432±20.7453 | 79.5227±14.4885 | 78.8345±14.6889 | 3.19E-03 | 2.98E-02 |
| 19 | Rhamnose | Carbohydrates | 0.7355±0.4350 | 1.5253±1.0684 | 1.2412±0.4800 | 3.40E-03 | 3.01E-02 |
| 20 | Pyroglutamic acid | Amino Acids | 23.2273±19.1316 | 36.9590±22.3356 | 43.9414±28.4839 | 4.32E-03 | 3.48E-02 |
| 21 | 2-Hydroxy-3-methylbutyric acid | Fatty Acids | 9.7839±6.1869 | 25.9517±41.4583 | 24.6407±21.5688 | 4.35E-03 | 3.48E-02 |
| 22 | Phenylacetylglutamine | Amino Acids | 1.8140±3.0730 | 4.5671±4.9836 | 5.8844±5.5653 | 5.85E-03 | 4.25E-02 |
| 23 | **Imidazolepropionic acid** | Imidazoles | 0.7613±0.0129 | 0.7649±0.0127 | 0.7914±0.0335 | 6.01E-03 | 4.25E-02 |
| 24 | Leucine | Amino Acids | 79.3103±51.7794 | 126.6939±124.3988 | 107.4868±24.4072 | 6.07E-03 | 4.25E-02 |
| **Supplementary Table 1 continued.** List of differentially expressed metabolites between HC, CN and CI groups. | | | | | | | |
| NO. | Metabolite | Class | HC (mean±SD) | CN (mean±SD) | CI (mean±SD) | Value of *p* | FDR |
| 25 | Threonic acid | Carbohydrates | 3.8588±1.8540 | 3.4587±2.4511 | 2.2624±1.2504 | 7.31E-03 | 4.92E-02 |
| 26 | **Homoserine** | Amino Acids | 5.2037±2.0715 | 6.0262±2.1035 | 7.7902±2.3761 | 7.94E-03 | 5.13E-02 |
| 27 | Malic acid | Organic Acids | 2.8439±1.4161 | 3.9973±1.7910 | 4.7079±2.2987 | 9.33E-03 | 5.81E-02 |
| 28 | N-Acetylneuraminic acid | Carbohydrates | 0.9681±0.3353 | 1.1854±0.3162 | 1.2658±0.4771 | 1.08E-02 | 6.30E-02 |
| 29 | Methylcysteine | Amino Acids | 5.0180±2.6655 | 7.4308±3.5565 | 9.7516±7.4175 | 1.09E-02 | 6.30E-02 |
| 30 | 4-Hydroxyproline | Amino Acids | 7.1767±3.5976 | 8.9286±3.3430 | 11.1255±4.8013 | 1.13E-02 | 6.32E-02 |
| 31 | Malonylcarnitine | Carnitines | 0.0499±0.0385 | 0.0782±0.0674 | 0.0949±0.0441 | 1.19E-02 | 6.45E-02 |
| 32 | Norleucine | Amino Acids | 9.8719±5.5238 | 14.6362±12.8089 | 14.2082±4.0191 | 1.23E-02 | 6.46E-02 |
| 33 | Isoleucine | Amino Acids | 102.2194±153.5211 | 91.5420±80.9166 | 95.0579±84.5616 | 1.33E-02 | 6.80E-02 |
| 34 | Kynurenine | Amino Acids | 1.0698±0.7853 | 1.5592±1.3256 | 1.7900±0.7251 | 1.41E-02 | 6.98E-02 |
| 35 | Oxoglutaric acid | Organic Acids | 11.5036±20.1530 | 17.6444±13.2115 | 16.5305±32.6802 | 1.62E-02 | 7.76E-02 |
| 36 | Succinic acid | Organic Acids | 2.1321±1.5120 | 2.4936±0.9362 | 3.8874±2.1266 | 2.35E-02 | 1.09E-01 |
| 37 | Dihomo-gamma-linolenic acid | Fatty Acids | 0.3885±0.4468 | 0.3454±0.1676 | 0.4453±0.2210 | 2.40E-02 | 1.09E-01 |
| 38 | Oleylcarnitine | Carnitines | 0.0743±0.0580 | 0.1609±0.2446 | 0.1585±0.1030 | 2.46E-02 | 1.09E-01 |
| 39 | Adrenic acid | Fatty Acids | 0.8479±1.5208 | 0.8115±0.5753 | 0.9805±1.1347 | 2.56E-02 | 1.10E-01 |
| 40 | Cystine | Amino Acids | 484.7222±291.8569 | 720.3711±298.9604 | 547.9601±234.7721 | 2.97E-02 | 1.25E-01 |
| 41 | Phenylacetic acid | Benzenoids | 3.6533±2.6299 | 31.7266±103.6197 | 8.5519±7.7915 | 3.27E-02 | 1.33E-01 |
| 42 | Phenyllactic acid | Phenylpropanoic Acids | 0.3029±0.2008 | 0.4576±0.1774 | 0.4597±0.2334 | 3.33E-02 | 1.33E-01 |
| 43 | Glutamine | Amino Acids | 984.6432±283.5144 | 1110.7761±287.8981 | 1227.5217±197.9785 | 3.47E-02 | 1.35E-01 |
| 44 | Indoleacetic acid | Indoles | 0.3525±0.3297 | 0.8676±1.3067 | 0.5541±0.2557 | 3.78E-02 | 1.44E-01 |
| 45 | Butyrylcarnitine | Carnitines | 0.0354±0.0307 | 0.0556±0.0446 | 0.1478±0.3431 | 4.11E-02 | 1.53E-01 |
| 46 | Creatine | Amino Acids | 43.7549±19.1291 | 49.3696±22.8996 | 61.5353±24.2558 | 4.95E-02 | 1.81E-01 |
| *FDR represents the p-value corrected. Mean represents the average relative abundance of metabolites in different groups, SD represents standard deviation. One-way ANOVA was used to compare the three groups, and p-value<0.05 is significant.* | | | | | | | |
